# Laparoscopic-assisted microwave ablation in treatment of small hepatocellular carcinoma: safety and efficacy in comparison with laparoscopic hepatectomy

**DOI:** 10.1186/s12893-024-02432-0

**Published:** 2024-05-07

**Authors:** Youping Wei, Lihong Zhang, Shun Zhang, Meina Song, Changhui Ji

**Affiliations:** 1 Department of imaging, Aviation General Hospital, China Medical University, Beijing, 100012 China; 2 Department of hepatobiliary surgery, Affiliated Hospital of Yangzhou University, Yangzhou, 225012 China; 3 Department of general surgery, Taixing Peoplès Hospital of Yangzhou University, No. 1, Changzheng Road, Taixing City, 225400 China

**Keywords:** Laparoscope, Laparoscopic hepatectomy, Microwave ablation, Small Hepatocellular Carcinoma

## Abstract

Laparoscopic-assisted microwave ablation (LAMWA), as one of the locoregional therapies, has been employed to treat hepatocellular carcinoma (HCC). This study aims to compare the efficacy and safety of LAMWA and laparoscopic hepatectomy in the treatment of small HCC.This study included 140 patients who met the inclusion criteria. Among them, 68 patients received LAMWA and 72 patients underwent laparoscopic hepatectomy. The perioperative condition, liver function recovery, the alpha fetoprotein (AFP) level, morbidities, hospitalization time, overall survival (OS), disease-free survival (DFS) and recurrence rate between the two groups were compared. The rate of complete elimination of tumor tissue was 100% and the AFP level was returned to normal within 3 months after surgery in both groups (*P* > 0.05). The mean alanine transaminase (ALT) and aspartate transaminase (AST) peak in the LAMWA group was lower than that in the laparoscopic hepatectomy group (259.51 ± 188.75 VS 388.9 ± 173.65, *P* = 0.000) and (267.34 ± 190.65 VS 393.1 ± 185.67, *P* = 0.000), respectively. The mean operation time in the LAMWA group was shorter than that in the laparoscopic hepatectomy group (89 ± 31 min VS 259 ± 48 min, *P* = 0.000). The blood loss in the LAMWA group was less than that in the laparoscopic hepatectomy group (58.4 ± 64.0 ml VS 213.0 ± 108.2 ml, *P* = 0.000). Compared with the laparoscopic hepatectomy group, patients in the LAMWA group had lower mean hospital stay (4.8 ± 1.2d VS 11.5 ± 2.9d, *P* = 0.000). The morbidities of the LAMWA group and the hepatectomy group were 14.7%(10/68) and 34.7%(25/72), respectively (*P* = 0.006). The one-, three-, and five-year OS rates were 88.2%, 69.9%, 45.6% for the LAMWA group and 86.1%, 72.9%, 51.4% for the laparoscopic hepatectomy group (*P* = 0.693). The corresponding DFS rates for the two groups were 76.3%, 48.1%, 27.9% and 73.2%, 56.7%, 32.0% (*P* = 0.958). Laparoscopic-assisted microwave ablation is a safe and effective therapeutic option for selected small HCC.

## Introduction

Hepatocellular carcinoma (HCC), the most common primary liver cancer, is one of the most prevalent malignant diseases worldwide, and the third most common cause of cancer-related deaths [1]. More than 700 000 cases worldwide are diagnosed yearly^[ 2]^. For small HCC (single HCC ≤ 3 cm or up to two nodules, each < 3 cm), liver transplantation is considered to be the best treatment. However, the scarcity of donors limits its application [3]. Laparoscopic hepatectomy is widely accepted as first line treatment for patients with small HCC and well-preserved liver function [4–6]. However, a significant concern remains in the trauma and postoperative complications that often accompany treatments. It is imperative to seek alternative yet effective therapeutic options that can minimize these adverse effects. Thanks to advancements in biomedical technology, microwave ablation has emerged as a crucial treatment modality for small HCC. Clinically, two commonly utilized methods are percutaneous microwave ablation guided by imaging techniques and laparotomy-assisted microwave ablation. Nevertheless, percutaneous microwave ablation can be challenging for tumors located in specific positions within the liver, often leading to a substantial increase in morbidity rates [7, 8]. Additionally, laparotomy-based microwave ablation may not be suitable for patients with compromised general health or dysfunction of vital organs, as it can significantly exacerbate surgical trauma [9, 10].

In view of the disadvantages of the two aforementioned microwave ablation methods, laparoscopic-assisted microwave ablation can partly compensate for theses disadvantages. A few reports have shown that.

LAMWA was as effective and safe as Radio Frequency Ablation (RFA) in treating small HCC [11, 12]. However, there are no studies comparing LAMWA and laparoscopic hepatectomy. The aim of this study is to compare the efficacy and safety of LAMWA and laparoscopic hepatectomy in the treatment of small HCC.

## Patient and methods

This study complies with the Helsinki Declaration and was approved by the Ethics Committee of the Aviation General Hospital of China Medical University, and the informed consent of the subjects was exempted. The approval number is HK2022-37.The study was an analytical study in 140 patients from August 2013 to October 2018 in the Aviation General Hospital of China Medical University. Among them, 68 for LAMWA and 72 for laparoscopic hepatectomy. The average age were 43.4 ± 12.8 years old in the LAMVA group, and 45.7 ± 11.9 years old in the laparoscopic hepatectomy group. The inclusion criteria [13] were: (i) single HCC ≤ 3 cm or up to two nodules, each < 3 cm; (ii) no extrahepatic metastasis or obvious vascular and intrahepatic bile ducts invasion; (iii) liver function of Child-Pugh Class A or B; (iv) no previous or simultaneous malignancies; (v) no previous treatment of HCC. The exclusion criteria were: (i) patients with Child–Pugh Class C or evidence of hepatic decompensation, including refractory ascites, esophageal or gastric variceal bleeding, or hepatic encephalopathy; (ii) patients with severe coagulation disorders (prothrombin time prolongation > 5s).

### Laparoscopic-assisted microwave ablation

**Fig. 1:**
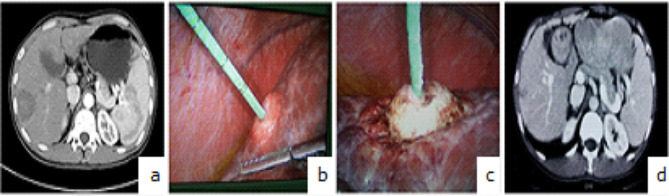
One case treated by laparoscopic-assisted microwave ablation. **a**: preoperative CT scan, **b**: antenna inserted into the center of the tumor under laparoscopy, **c**: the tumor coagulated by microwave, **d**: CT scan showed the tumor inactivated by microwave

The ECO-100 series cool cyclic microwave (Yigao medical equipment, Inc., Nanjing, China) was used. The parameter settings were as follows [14]: power: 220 V, the frequency: 2450 MHz, the output power: 55–60 W, and the ablation time: 5–10 min. The patient is placed in different positions such as lying flat, lateral recumbency, prone position, or a tilted position with the head lower than the feet, in order to facilitate the optimal observation angle and treat the tumor in the liver. Under general anesthesia, a supine position with the head side raised by 20 degrees was often used. After anesthesia was achieved, a 15-cm long 14-gauge unipolar cooled-shaft antenna was inserted into the center of the tumor. The whole procedure was guided and constantly monitored by ultrasound (Aloka 5000, Tokyo, Japan) with 1–5 MHz convex array probes. The number of repetitions of thermal ablation varied depending on the different sizes, shapes, and location of the tumor as well as the coagulation effects. The ablation time was over when the real-time ultrasound demonstrated that the entire tumor and a surrounding 1-cm safety margin were enveloped by hyperechoic microbubbles. At the end of the procedure, the needle track was coagulated to prevent bleeding. (Fig. 1)

### Laparoscopic hepatectomy [15]

The patient lay on their back with their head high and feet low. According to the location of the tumor, the operating table was tilted to the left or right. The pneumoperitoneum pressure was controlled at 12–15 mmHg. Five trocars were placed in a fan-shaped distribution around the tumor. Firstly, an investigation was conducted on the condition of the liver and tumor, as well as the presence or absence of metastasis. During the surgery, the tumor location was confirmed by ultrasound and the tangent line was marked with ultrasound assistance. Surgical technique and hepatic blood flow blockade method were determined based on the tumor condition. The harmonic scalp was used to dissect the perihepatic ligament. Starting from the surface of the liver, the liver tissue was gradually dissected by using electric and harmonic scalp until the liver tumor was completely removed. Blood vessels and bile ducts were cut off after being clamped with titanium or hemlock clips. The hepatic cross section was treated with bipolar electrocoagulation for the bleeding site. 3 − 0 or 4 − 0 absorbable suture was used for active bleeding.The liver section was washed repeatedly with sterile distilled water to confirm that there was no obvious active bleeding or bile leakage. To remove the specimen, the incision was enlarged through the abdominal operation hole or the transverse incision above the pubic symphysis. The definition of R0 resection is the absence of tumor invasion at the surgical resection edge under the microscope. In fact, it is known that the growth of HCC in cirrhotic livers can be interdigitated and in one part of the section the limit may be 1 cm but in another 1 mm or even in contact with the margin in clinical work.

LAMWA and laparoscopic hepatectomy were performed by the same experienced surgeon and his teams. Patients in both groups remained hospitalized until liver functions approached normal and complications resolved. The perioperative condition, liver function, morbidities and hospitalization time were recorded.

### Follow up

The primary study endpoint is Overall Survival (OS), defined as the time elapsed until death due to any cause. Meanwhile, Disease-Free Survival (DFS) serves as a secondary study endpoint, encompassing the time until either recurrence or death occurs, whichever comes first. Patients were reviewed at 1 month and sequential 3 month intervals post-procedure. Contrast-enhanced computed tomography (CT) was used to evaluate the effectiveness at the first month after procedure. Serum AFP and ultrasound were monitored every 3 months, and contrast enhanced CT was done every 6 months. The tumor recurrence was evaluated by CT or magnetic resonance imaging. The data of both groups were collected by means of outpatient, inpatient and telephone. All the patients were followed up for more than 36 months after operation.

Statistical analysis Data was analyzed by using SPSS software, version 22.0 (SPSS Inc., Chicago, IL, USA). Significance was set at *P* < 0.05.For continuous variables, Student’s t-test and Mann–Whitney U-test were applied. For categorical variables, χ2 test and Fisher exact test were performed. The OS curves, DFS curves, and overall recurrence curves were generated by the Kaplan–Meier method and compared by log–rank test.

## Results

Baseline characteristics were not significantly different between the two groups (Table 1). In the LAMWA group, assessed by contrast-enhanced CT for 1 month after the initial treatment, 79 lesions in 68 patients were completely ablated with a 0% conversion rate to an open procedure. For the laparoscopic hepatectomy group, partial resection, wedge resection, lobectomy and hemi-hepatectomy were done in 56, 8, 4 and 4 patients, respectively. All of these patients had at least 1–2 cm tumor-free resection margins. All of them received R0 resection.

**Table 1:** Clinical characteristics of patients in the two groups

	LAMVA (*n* = 68)	laparoscopic hepatectomy (*n* = 72)	*P* value
Age (year)	43.4 ± 12.8	45.7 ± 11.9	0.273
Gender (male/female)	52/16	58/14	0.556
Childs class:			
A/B/C	55/13/0	58/14/0	0.961
Cirrhosis (+/−)	56/12	60/12	0.878
Liver function:			
Total bilirubin (µmol/L)	11.07 ± 5.85	12.13 ± 6.37	0.308
Prothrombin time (s)	11.35 ± 5.38	12.05 ± 5.47	0.447
Serum albumin (g/L)	41.58 ± 12.41	40.69 ± 13.51	0.686
ALT(U/L)	33.14 ± 23.15	35.22 ± 22.48	0.591
AST(U/L)	36.32 ± 25.62	38.28 ± 27.38	0.663
HBsAg (+), n	61	67	0.479
AFP ( ng/ml)	178.1 ± 78.6	181.5 ± 88.3	0.798
Tumor size (cm)	2.5 ± 1.7	2.6 ± 1.6	0.721
Tumor number (1/2)	60/8	65/7	0.696

Note: ALT: alanine transaminase, AST: aspartate transaminase, AFP: alpha fetoprotein

Average operative time for LMWA was 89 ± 31 min versus 259 ± 48 min for laparoscopic hepatectomy (*P* = 0.000). The average estimated blood loss for LMWA was 58.40 ± 64 ml versus 213 ± 108.2 ml for laparoscopic hepatectomy (*P* = 0.000). Ten cases of complications in the LMWA group, including 3 cases of pleural effusion, 1 case of abdominal bleeding, 3 cases of peritoneal effusion, 1 case of Grade A bile leakage and 2 cases of surgical site infection. Twenty five patients had surgical related complications in laparoscopic hepatectomy, including 5 cases of pleural effusion, 3 cases of abdominal bleeding, 12 cases of peritoneal effusion, 1 case of Grade A and 2 cases of Grade B bile leakage and 2 cases of surgical site infection. Based on the varying conditions of the complications, treatments such as drainage and hemostasis with medication were adopted, and all complications were improved. The morbidities in the LAMWA group and laparoscopic hepatectomy group were 14.7%(10/68) and 34.7%(25/72), respectively, with a significant difference between the two groups(*P* = 0.006). The complications were classified according to Clavien Dindo system [16], and most of them were in grade I and II, which were cured through medication or blood transfusion. Two cases of grade III complications in the LAMWA group. One case with a large amount of pleural effusion was cured through intercostal tube drainage. One case of bile leakage resulting in fever and abdominal pain, was cured after sonar guided tube drainage. Three cases of grade III complications in the laparoscopic hepatectomy group. One case with a large amount of pleural effusion was cured through thoracic puncture and tube drainage. After performing puncture and tube drainage, two instances of Grade B bile leakage resulting from inadequate drainage through the initial abdominal drainage tube were successfully resolved. There were no death and severe complications(Clavien Dindo > III) in both groups. The average hospitalization time was 6.8 ± 2.1 days in LAMVA group, while 11.5 ± 2.9 days in HR group. There was significant difference between the two groups (*p* = 0.000). (Table 2)

**Table 2:** Comparison of operation situation, complications and hospitalizing period between two groups

	LAMVA (*n* = 68)	laparoscopic hepatectomy (*n* = 72)	*P* value
Average operative times (min)	89 ± 31	259 ± 48	0.000
Average blood loss (ml)	58.40 ± 64	213.0 ± 108.2	0.000
Average hospitalization time(d)	4.8 ± 1.2	11.5 ± 2.9	0.000
Complication, n	10(14.7%)	25(34.7%)	0.006
Pleural effusion	3	5	0.519
Abdominal bleeding	1	3	0.339
Peritoneal effusion	3	12	0.022
Biliary fistula	1	3	0.339
Surgical site infection	2	2	0.954
Death	0	0	1.000
Clavien-Dindo classification(n)			0.006
I	5	12	
II	2	9	
III	2	3	
IV	0	0	
V	0	0	

The serum AFP level was returned to normal within 3 months in both groups. No significant difference of decline rate was found (*P* > 0.05). The mean ALT and AST peak in LAMWA group was lower than that in laparoscopic hepatectomy group with significant differences (259.51 ± 188.75 VS 388.9 ± 173.65, *P* = 0.000) and (267.34 ± 190.65 VS 393.1 ± 185.67, *P* = 0.000), respectively. (Table 3)

**Table 3:** Comparison of the liver function and serum AFP level between two groups

	LAMVA (*n* = 68)	laparoscopic hepatectomy (*n* = 72)	*P* value
Liver function:			
ALT peak (U/L)	259.51 ± 188.75	388.9 ± 173.65	0.000
AST peak (U/L)	267.34 ± 190.65	393.1 ± 185.67	0.000
AFP(ng/ml)			
1 month after operation	56.1 ± 52.9	63.3 ± 60.2	0.870
2months after operation	26.8 ± 20.8	28.5 ± 21.6	0.788
3 months after operation	6.2 ± 3.2	5.8 ± 2.9	0.439

**Fig. 2:**
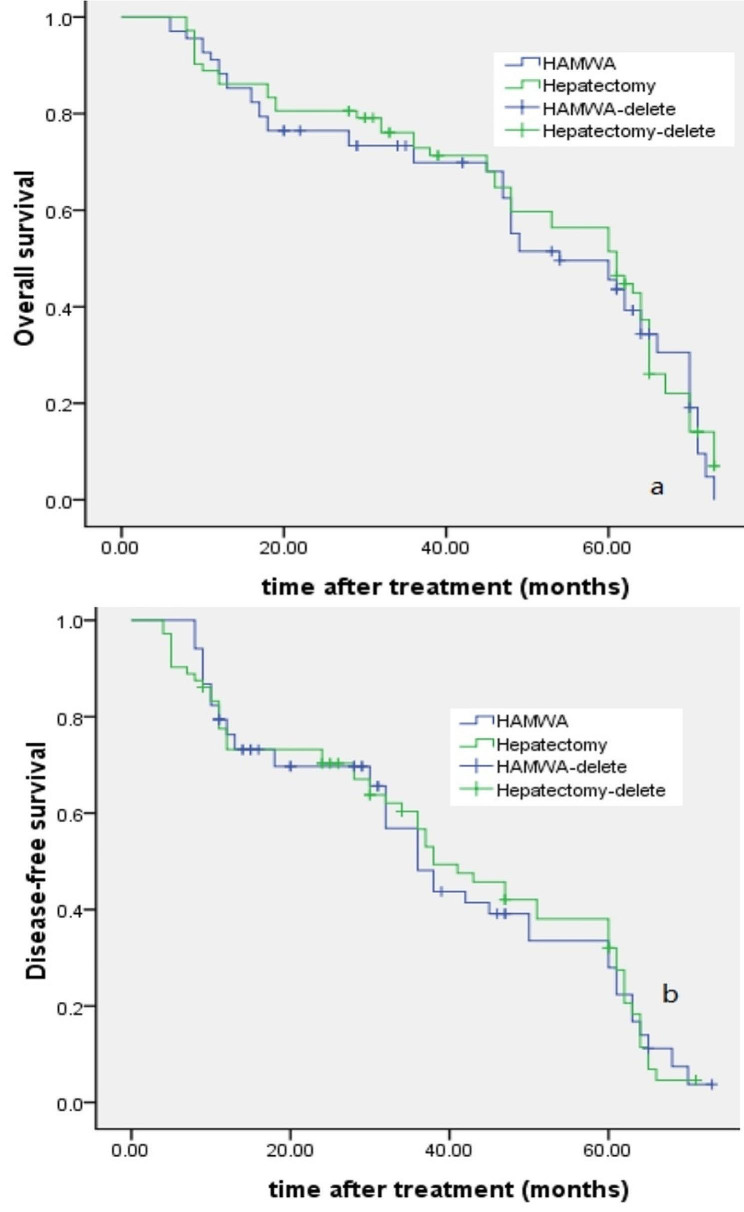
Survival and recurrence curves of small hepatocellular carcinoma (HCC) patients who underwent laparoscopic-assisted microwave ablation (LAMWA) and laparoscopic hepatectomy. **(a)** Overall survival did not differ significantly between the two groups. **(b)** Disease-free survival had no significance between the two groups

The median follow-up time was 42.9 (36–74) months for the MWA group, and 44.2 (38–73) months for the laparoscopic hepatectomy group. The 1-, 3-, and 5-year OS rates were 88.2%, 69.9%, 45.6% for the LAMWA group and 86.1%, 72.9%, 51.4% for the laparoscopic hepatectomy group (χ2 = 0156, *P* = 0.693). The corresponding DFS rates for the two groups were 76.3%, 48.1%, 27.9% and 73.2%, 56.7%, 32.0% (χ2 = 0.003, *P* = 0.958). (Fig. 2).

## Discussion

Over the years, liver resection has been the preferred method for hepatocellular carcinoma and currently is recognized as the best treatment for its long-term curative effect [17–19]. However, patients suffering from liver dysfunction are at a heightened risk of experiencing trauma from laparoscopic hepatectomy. Furthermore, the presence of abdominal adhesions resulting from laparoscopic hepatectomy can significantly exacerbate the challenges associated with reoperation or liver transplantation in cases of tumor recurrence. Additionally, the complications associated with the surgery and the extended hospital stay can influence some patients’ decision to opt for hepatectomy. Consequently, with the objective of safeguarding therapeutic efficacy, there is a growing trend towards selecting treatment methods that minimize injury, pain, and expedite recovery. Multiple locoregional therapies, including microwave ablation, radiofrequency ablation, etc., are a safe, minimally invasive, and effective treatment for hepatocellular carcinoma (HCC) [20–23], and microwave ablation has been developed rapidly in recent years [24–28].

Microwave energy, conducted to an electrode, penetrated a few centimeters into the tissue and caused the tissue to generate heat by changing the polarity of the water molecules. When the temperature gets up to 60℃, denaturation and solidification of tumor cells take place, which results in tumor cells irreversible necrosis [29, 30]. Many reports indicated that there was no significant difference of the therapeutic effect especially for small liver cancer between microwave ablation and surgical resection [31–33].

At present, CT or ultrasound was generally used to guide the microwave ablation. Past experience showed that the accuracy of percutaneous puncture was affected by breathing and adjacent cavity organ, eventually leading to the lesion completely inactivated. In some cases, although the tumor size was not big, in order to inactivate the tumor more accurately and completely, repeated electrode insertion and irradiation were required to obtain a sufficiently large treated area, which may increase the chances of tumor cells implantation and metastasis along the needle tract [8]. In addition, especially for the tumor at the dome of the liver or visceral surface adjacent to the gallbladder, kidney and duodenum and great vessels, or multiple tumors, it is difficult to perform percutaneous microwave ablation. For some cases who cannot tolerate laparotomy and at the same time the tumors were located in the special parts of the liver, such as diaphragm or lower part, imaging guided percutaneous puncture faced serious complication risk [7].

In view of this, LAMWA as a new technology for HCC is a combination of laparoscopic and microwave treatment. Our study showed that, compared with laparoscopic hepatectomy, LAMWA had shorter operation time, less bleeding, fewer complications, high security, less damage to postoperative liver function and short hospitalization time. Laparoscopic puncture is under direct vision, has excellent visualization, and the surrounding tissue and adhesion site can be retracted or loosened, which reduces the occurrence rate of serious complications, such as bleeding, bile leakage, and pneumothorax etc. Due to its minimal injury and reduced abdominal adhesions, it establishes favorable conditions for reoperation or liver transplantation in cases of recurrence. All of 68 patients were well tolerated with the microwave ablation. There were no cases of bile leakage, bleeding, perforation of gastrointestinal tract, gallbladder heart reflection and serious complications such as pneumothorax due to diaphragmatic injury.

As reported, for patients with solitary HCC ≤ 3 cm, the 5-year OS rates after laparoscopic hepatectomy were ranged from 66.9 to 74.6%, the 5-year DFS rates from 26.0 to 36.9% [23, 34]. The results of our study were consistent with published studies. With microwave ablation, Takami Y et al.^27^ reported that the 1-, 3-, and 5-year OS rates were 97.9%, 85%, and 70% for a single small HCC. Shi J et al. [35] reported that the1-, 3-, and 5-year DFS rates were85%, 54% and 33%. Our study showed that the 1-, 3-, and 5-year OS and DFS rates were in accordance with the results in the two aforementioned studies. We did not find any significant differences in OS and DFS when comparing LAMWA and laparoscopic hepatectomy. The current study also found that the 1-, 3- and 5-year recurrence rates in the two groups were similar. A large safety margin is one of the important factors that influence long-term effects for small HCC after curative treatments. Some studies have shown that under microscope, tumors usually exceed the macroscopic boundary by more than 1 cm [36, 37]. In our study, for patients received laparoscopic hepatectomy, at least 1–2 cm tumor free margins were got. Meanwhile, LAMWA with our instrument can produce a necrotic area about more than 4 cm in diameter in one session, allowing full ablation of a less than 3-cm tumor plus a 1.0-cm tumor free margin [38]. Puncture needle is imported through the Trocar, repeated puncture with multiple lesions, multi angle and multi direction can be carried out at one site to decrease the possibility of tumor metastasis in the abdominal needle tract. The range of microwave ablation is clearly visible, and ablation time can be adjusted at any time according to the actual effect, which can reduce the possibility of residual of tumor. Thus, the long-term therapeutic effects of the two treatments were similar.

There were some limitations in our study. First, the small sample size of patients decreased the statistical strength and led to bias. Second, this was a single-center, retrospective study with all its inherent defects. Multicenter, prospective, and large randomized controlled trials should be performed to confirm these results.

## Conclusions

Our study suggests that compared with laparoscopic hepatectomy, LAMWA had shorter operation time, less bleeding, fewer complications, high security, less damage of postoperative liver function and short hospitalization time during the perioperation period. As for long-term effect, LAMWA had a similar effects of 1-, 3-, and 5-year OS, DFS and recurrence rate compared with laparoscopic hepatectomy. LAMWA is a safe and effective therapeutic option for selected small HCC.

## Data Availability

“The datasets used and/or analysed during the current study available from the corresponding author on reasonable request.”
